# Behaviour and epidemiology of differentiated thyroid cancer among filipinos in and outside the Philippines: Comparison between Qatar, Canada and Philippines

**DOI:** 10.1016/j.amsu.2022.104202

**Published:** 2022-07-18

**Authors:** Mohamed Said Ghali, Walid El Ansari, Abdelrahman Abdelaal, Mohamed S. Al Hassan

**Affiliations:** a Department of Surgery, Hamad General Hospital, Hamad Medical Corporation, Doha, Qatar; b Department of General Surgery, Ain Shams University, Cairo, Egypt; c College of Medicine, Qatar University, Doha, Qatar; d Weill Cornell Medicine – Qatar, Doha, Qatar

**Keywords:** Thyroid, Neoplasms, Cancer, Follicular, Papillary, Filipino, Qatar

## Abstract

**Background:**

No study assessed the behaviour/epidemiology of differentiated thyroid cancer (DTC) among Filipinos in the Middle East/north Africa; or compared such behaviour with Filipino DTC patients in and outside the Philippines.

**Materials and methods:**

Retrospective review of all adult Filipinos with DTC diagnosed between 2015 and 2020 at our institution [papillary TC (PTC, n = 110); follicular TC (FTC, n = 4)]. Chi-square or *t*-test compared the frequency, demography, history, ultrasound, laboratory and histopathology of PTC vs FTC; and compared the epidemiology of DTC among Filipinos in Qatar vs Canada (1 study) vs the Philippines (3 studies).

**Results:**

DTC frequency was ≈43.84 cases/100,000 Filipinos in Qatar. Males had more aggressive disease in terms of lymphovascular invasion, number of nodules, stage IV disease and metastases. PTC and FTC were similar, but more PTC patients presented with solid tumors and higher number of involved lymph nodes. DTC behaviour among Filipinos in Qatar vs Canada was fairly homogenous. Conversely, DTC among Filipinos in Qatar vs Philippines exhibited many dissimilarities: Qatar had significantly younger patients, less hyperthyroid/thyrotoxicity symptoms and neck swelling at presentation, fewer solid nodules, micro-calcifications, and multi-nodular disease; smaller tumors; more stage I and less stage IV disease; and no distant metastasis (*P* 0.01 to < 0.0001).

**Conclusions:**

DTC epidemiology among Filipinos in Qatar and Canada was fairly homogenous. Conversely, DTC in Qatar vs the Philippines was dissimilar. DTC among Filipinos in Qatar has presentations that could be misinterpreted as non-suspicious. This warrants additional practices for earlier detection among Filipinos.

## Highlights

• Epidemiology of differentiated thyroid cancer among Filipinos living Qatar vs Canada has considerable similarities.
• Epidemiology of differentiated thyroid cancer among Filipinos living Qatar vs the Philippines appears to be heterogenous.
• Differentiated thyroid cancer among Filipinos living in Qatar has presentations that could erroneously be misinterpreted as non-malignant by unsuspecting health worker, and seemingly lower tumor load compared to those living in the Philippines.
• Thyroid lesions among Filipinos in Qatar should be taken seriously, warranting additional practices for earlier detection among Filipinos.

## 1 Introduction

Papillary thyroid cancer (PTC) is the most common type of differentiated thyroid cancer (DTC), accounting for >80% of thyroid cancers, whereas follicular thyroid cancer (FTC) is the second most common (10–15%) [[1], [2], [3]]. The frequency of DTC has risen in the past decades [2]. Generally, thyroid cancer has low mortality rates and favorable prognosis [4,5]. However, some racial/ethnic populations display a higher likelihood e.g., Filipinos exhibit one of the highest thyroid cancer rates [6]. An important focus of thyroid cancer research has been to ascertain the factors associated with poor disease outcomes [[7], [8], [9]].

Generally, the distribution of health of different ethnic groups varies by region of origin, region of destination and their combination [10]. Despite this, most studies of thyroid cancer among Filipinos examined those living in the Philippines [2,11,12]. The very few studies that appraised thyroid cancer among Filipinos living outside the Philippines were mostly in USA or Canada [7,13,14]. To our knowledge, no studies assessed thyroid malignancy among Filipinos living in the Middle East and North Africa (MENA) region, despite that countries of the Arabian Gulf are among the top 10 destinations for Filipinos [15]. There is a lack of thyroid cancer studies that assessed the association between thyroid cancer and country, with calls for analysis of thyroid cancer across different regions of the world [16].

Therefore, the current study bridges this knowledge gap to examine the epidemiology, clinical presentation, features and characteristics of DTC among Filipinos in Qatar. The specific objectives were to assess the:

● Frequency of PTC and FTC;
● Demographic, history and surgical characteristics; and,
● Ultrasonographic, pre-op laboratory, post-op histopathology, and management features.

In addition, we compared the above findings with those of Filipino patients with DTC living in the Philippines and elsewhere outside the Philippines using published articles identified after a literature search. To the best of our knowledge, the current study is the first to undertake such a task. The findings add to the thin evidence base on the subject.

## 2 Methods

### 2.1 Study design, ethics and procedures

Filipinos represent the third largest group of expatriates in Qatar [17]. Hamad General Hospital is the largest tertiary care facility in Qatar and receives all the thyroid cancer cases in the country. The Medical Research Center (IRB) of Hamad Medical Corporation approved this retrospective study (#MRC-01-20-983). The inclusion criteria included all Filipinos ≥18 years diagnosed with DTC between May 2015–June 2020 (N = 114). Using patients’ hospital case numbers, we retrieved three sets of pre- and post-operative data from the electronic records database. These included demographic, history and surgical characteristics; ultrasonographic and FNA (fine needle aspiration) findings, pre-op laboratory, and histopathology data; and the use/doses of radioactive iodine ablation (RAI) or external beam radiotherapy, recurrence, and survival. Staging of thyroid cancer used the AJCC-8 staging system for patients diagnosed during 2018–2020; and AJCC-7 for patients diagnosed during 2015–2017 [18,19]. The AJCC/TNM system predicts survival among patients with cancer [9,20].

### 2.2 Literature search for articles to compare with the current study

Using PubMed, MEDLINE, Scopus, and Google scholar electronic databases, we searched the literature for studies of Filipino patients with DTC living in and outside the Philippines published between January 2005 to March 2022. Additional searches were performed employing the reference lists of studies. After examining the abstracts and full texts, the research team selected 4 suitable papers, 3 in the Philippines [11,21,22] and 1 in Canada [7]. Data pertaining to three sets of pre/post-operative variables under examination were extracted from the 4 selected articles, tabulated and compared to the findings from Qatar. We report the current study in line with the STROCSS 2021 criteria [23], with research registry unique identifying number [24].

### 2.3 Statistical analysis

Data were presented as mean ± standard deviation (SD) or frequency and percentage as appropriate. Using chi-square for categorical variables and *t*-test for continuous, PTC was compared FTC; and findings of the present study were compared to other studies in the Philippines and Canada. Data analysis used the Statistical Package for Social Sciences version 21 (SPSS Inc., Chicago, IL), and online statistical package OpenEpi [25], with significance set at *P* < 0.05.

## 3 Results

Across our 114 patients, PTC and FTC comprised 96.5% and 3.5% respectively. Common PTC variants included classic (74.6%) and follicular (13.2%) variants. The 114 diagnosed cases divided by the 260,000 Filipinos in Qatar during the study period [17] generated an incidence of ≈43.84 DTC cases per 100,000 Filipinos living in Qatar.

### 3.1 Clinical, ultrasonographic, FNA and laboratory profile

Table 1 shows that mean age was 40.15 years, and females were overrepresented (83.3%). The only significant differences between PTC vs FTC patients was that surgically, PTC was associated with more total thyroidectomy + lymph node dissection or right hemi-thyroidectomy; and for ultrasound (US), more PTC patients presented with solid tumors, while FTC patients presented more with mixed solid/cystic tumors. PTC and FTC patients displayed significantly different Bethesda scores (Table 2), but did not significantly differ across the laboratory characteristics (Table 3).

**Table 1:** Clinical profile of Filipino patients with differentiated thyroid cancer.

Characteristic	Whole sample (N = 114)	PTC (*n* = 110, 96.5%)	FTC (*n* = 4, 3.5%)	*p-*value
Demography				
Sex				0.36
Male	19 (16.7)	19 (17.3)	0 (0)	
Female	95 (83.3)	91 (82.7)	4 (100)	
Age (years, M±SD)	40.15 ± 9.86	40.25 ± 9.94	37.5 ± 7.9	0.59
Age bracket (years)				0.80
< 45	79 (69.3)	76 (69.1)	3 (75)	
≥ 45	35 (30.7)	34 (30.9)	1 (25)	
History				
Head and neck radiation therapy (no)	114 (100)	110 (100)	4 (100)	**—**
Voice change (yes)	6 (5.4)	5 (4.6)	1 (25)	0.20
Weight loss (yes)	3 (2.7)	3 (2.8)	0 (0)	1
Neck swelling (yes)	88 (78.6)	85 (78.7)	3 (75)	1
Thyrotoxicity (yes)	6 (5.4)	5 (4.7)	1 (25)	0.20
Family history of thyroid cancer (yes)	5 (4.4)	5 (4.5)	0 (0)	1
Initial surgical approach				*0.002*
Type				
Total thyroidectomy	64 (56.1)	62 (56.4)	2 (50)	
Left hemi-thyroidectomy	7 (6.1)	5 (4.5)	2 (50)	
Right hemi-thyroidectomy	11 (9.6)	11 (10)	0 (0)	
Total thyroidectomy + neck dissection	32 (28.1)	32 (29.1)	0 (0)	
Complication/s				0.99
No complications	105 (92.9)	101 (92.7)	4 (100)	
Bleeding	1 (0.9)	1 (0.9)	0 (0)	
Right RLN injury	2 (1.8)	2 (1.8)	0 (0)	
Left RLN injury	1 (0.9)	1 (0.9)	0 (0)	
Bilateral RLN injury	0 (0)	0 (0)	0 (0)	
Parathyroid injury	3 (2.7)	3 (2.8)	0 (0)	
RLN + parathyroid injury	1 (0.9)	1 (0.9)	0 (0)	

Values expressed as frequency (%) except where stated otherwise; M±SD mean ± standard deviation. *PTC* papillary thyroid cancer; *FTC* follicular thyroid cancer; *RLN* recurrent laryngeal nerve; — not applicable. Italicized cells indicate statistical significance.

**Table 2:** Ultrasonographic and FNA profiles of Filipino patients with differentiated thyroid cancer.

Characteristic	Whole sample (N = 114)	PTC (*n* = 110, 96.5%)	FTC (*n* = 4, 3.5%)	*p-*value
US				
Thyroid Nodules				1
Uninodular	55 (49.1)	53 (49.1)	2 (50)	
Multinodular	57 (50.9)	55 (50.9)	2 (50)	
Aspect				*0.025*
Cystic	6 (5.5)	6 (5.7)	0 (0)	
Mixed solid/cystic	23 (20.9)	20 (18.9)	3 (75)	
Solid	81 (73.6)	80 (75.5)	1 (25)	
Maximum size (cm, M±SD)	2.60 ± 1.32	2.59 ± 1.32	2.98 ± 1.61	0.57
Micro-calcification				0.53
Yes	19 (17.1)	18 (16.8)	1 (25)	
No	92 (82.9)	89 (83.2)	3 (75)	
FNA (Bethesda score)				*0.02*
I	1 (0.9)	1 (1)	0 (0)	
II	16 (14.7)	15 (14.3)	1 (25)	
III	28 (25.7)	27 (25.7)	1 (25)	
IV	2 (1.8)	1 (1)	1 (25)	
V	14 (12.8)	14 (13.2)	0 (0)	
VI	48 (44.1)	47 (44.8)	1 (25)	

Values expressed as frequency (%) except where stated otherwise; M±SD mean ± standard deviation. *PTC* papillary thyroid cancer; *FTC* follicular thyroid cancer; FNA (fine needle aspiration) Bethesda score [I Nondiagnostic/Unsatisfactory, II Benign, III Atypia or FLUS (Follicular lesion of undetermined significance), IV Follicular Neoplasm or Suspicious for a Follicular Neoplasm, V Suspicious for Malignancy, VI Malignant]; Italicized cells indicate statistical significance.

**Table 3:** Laboratory profile of Filipino patients with well-differentiated thyroid cancer.

Characteristic	Whole Sample (N = 114)	PTC (*n* = 110, 96.5%)	FTC (*n* = 4, 3.5%)	*p-*value
Pre-operative (reference range)				
Ca (2.10–2.60 mmol/L)	2.31 ± 0.12	2.31 ± 0.12	2.25 ± 0.09	0.37
Free T3 a (3.7–6.4 pmol/L)	4.24 ± 1.20	4.17 ± 1.24	5.02 b	0.53
Free T4 (11–23.3 pmol/L)	13.78 ± 3.06	13.77 ± 3.08	13.99 ± 2.68	0.90
TSH (0.3–4.2 mIU/L)	2.38 ± 3.99	2.41 ± 4.05	1.29 ± 1.56	0.63
WBC (4–10 μL)	7.73 ± 2.13	7.76 ± 2.15	8.33 ± 0.81	0.62
Hb (12–15 gm/dL)	13.34 ± 1.60	13.39 ± 1.61	11.93 ± 0.38	0.12
PLT (150–400 μL)	304.61 ± 71.06	303.69 ± 71.2	334 ± 72.54	0.47
Lymphocyte (1–3 μL)	2.59 ± 3.20	2.60 ± 3.25	2.27 ± 0.64	0.86
Neutrophil (2–7 μL)	5.20 ± 5.74	5.2 ± 5.83	5.2 ± 0.96	0.99
Post-operative				
TSH (suppression) (0.3–4.2 mIU/L)				
Median (range)	1.88 (0.01–99.2)	1.88 (0.01–99.2)	2.89 (0.01–39.5)	0.80
Tumour markers				
Thyroglobulin (<0.1 ng/mL)				
Median (range)	0.90 (0.07–8458.2)	0.90 (0.07–8458.2)	1.45 (0.1–19.7)	0.83
Thyroglobulin antibodies (≤22IU/mL)				
Median (range)	0.9 (0.1–1981)	0.9 (0.1–1981)	0.9 (0.9–91.7)	1

Values expressed as mean ± SD except where stated otherwise. PTC papillary thyroid cancer; *FTC* follicular thyroid cancer; Ca calcium; *T3* Triiodothyronine; *T4* Thyroxine; *TSH* thyroid stimulating hormone; *WBC* white blood cells; Hb hemoglobin; *PLT* platelets.

a Only 11 cases, as it is not routinely undertaken for all patients, with reliance on free T4 and TSH.

b No standard deviation as there was only one case.

### 3.2 Histologic profile, staging, management, and outcomes

PTC and FTC patients did not differ across the histopathological characteristics except that PTC cases had a slightly higher mean number of involved nodes (*P* = 0.004) (Table 4). PTC and FTC did not differ in the disease stages (Table 5) or in the management, recurrence and survival features (Table 6).

**Table 4:** Histologic profile of Filipino patients with differentiated thyroid cancer.

Characteristic	Whole sample (N = 114)	PTC (*n* = 110, 96.5%)	FTC (*n* = 4, 3.5%)	*p-*value
Tumor				
Size, (cm, M±SD)	2.31 ± 1.54	2.29 ± 1.55	2.82 ± 1.32	0.50
Size (cm)				0.58
< 4	94 (84.7)	91 (85)	3 (75)	
≥ 4	17 (15.3)	16 (15)	1 (25)	
Number (M±SD)	1.64 ± 0.87	1.65 ± 0.88	1.25 ± 0.50	0.37
Number of tumors				0.92
1	61 (55.5)	58 (54.7)	3 (75)	
2	33 (30)	32 (30.2)	1 (25)	
3	13 (11.8)	13 (12.3)	0 (0)	
4	2 (1.8)	2 (1.9)	0 (0)	
6	1 (0.9)	1 (0.9)	0 (0)	
Focality				0.24
Unifocal	60 (54.1)	59 (55.1)	1 (25)	
Multifocal	51 (45.9)	48 (44.9)	3 (75)	
Margin				0.23
Involved	29 (26.1)	29 (27.1)	0 (0)	
Not involved	82 (73.9)	78 (72.9)	4 (100)	
LV invasion				0.31
Yes	27 (24.8)	27 (25.5)	0 (0)	
No	82 (75.2)	79 (74.5)	3 (100)	
LN involvement				0.42
Yes	50 (44.6)	49 (45.4)	1 (25)	
No	62 (55.4)	59 (54.6)	3 (75)	
Number of involved LN (M±SD)	5.02 ± 4.77	5.02 ± 4.82	5.00 ^*a*^	*0.004*

Values expressed as frequency (%) except where stated otherwise; M±SD mean ± standard deviation. *PTC* papillary thyroid cancer; *FTC* follicular thyroid cancer; *LN* lymph node/s; *LV* lymphovascular. ^*a*^ No standard deviation as there was only one case; Italicized cells indicate statistical significance.

**Table 5:** Staging of Filipino patients with differentiated thyroid cancer.

TNM	Whole Sample (N = 114)	PTC (*n* = 110, 96.5%)	FTC (*n* = 4, 3.5%)	*p-*value
TNM				
T				0.68
T1a	29 (25.9)	29 (26.9)	0 (0)	
T1b	20 (17.9)	19 (17.6)	1 (25)	
T2	28 (25.0)	26 (24.1)	2 (50)	
T3a	33 (29.5)	32 (29.6)	1 (25)	
T3b	2 (1.8)	2 (1.9)	0 (0)	
T4	0 (0)	0 (0)	0 (0)	
N				0.440
NX	43 (37.7)	41 (38)	2 (50)	
N0	28 (24.6)	26 (24.1)	2 (50)	
N1a	20 (17.5)	20 (18.5)	0 (0)	
N1b	21 (18.4)	21 (19.4)	0 (0)	
M				**—**
M0	114 (100)	110 (100)	4 (100)	
M1	0 (0)	0 (0)	0 (0)	
AJCC Stage				0.87
I	97 (85.1)	93 (84.5)	4 (100)	
II	11 (9.6)	11 (10.0)	0 (0)	
III	4 (3.5)	4 (3.6)	0 (0)	
IV	2 (1.8)	2 (1.8)	0 (0)	

Values expressed as frequency (%). *PTC* papillary thyroid cancer; *FTC* follicular thyroid cancer; *TNM* tumor, nodes, metastases; — not applicable.

**Table 6:** Management, recurrence and survival characteristics of Filipinos with differentiated thyroid cancer.

Characteristic	Whole Sample (N = 114)	PTC (*n* = 110, 96.5%)	FTC (*n* = 4, 3.5%)	*p-*value
Management after surgery				
RAI				0.76
Yes	75 (74.3)	73 (74.5)	2 (66.7)	
No	26 (25.7)	25 (25.5)	1 (33.3)	
Dose				0.33
High	24 (32)	24 (32.9)	0 (0)	
Low	51 (68)	49 (67.1)	2 (100)	
Number of Doses				
High Dose				**—**
Mean ± SD	1.35 ± 0.65	1.35 ± 0.65	0 (0)	
Doses				**—**
1 dose	17 (73.9)	17 (73.9)	0 (0)	
2 doses	4 (17.4)	4 (17.4)	0 (0)	
3 doses	2 (8.7)	2 (8.7)	0 (0)	
Low Dose				
Mean ± SD	2.02 ± 0.89	2.06 ± 0.89	1 ± 0	0.1
Doses				0.22
1 dose	16 (32)	14 (29.2)	2 (100)	
2 doses	19 (38)	19 (39.6)	0 (0)	
3 doses	14 (28)	14 (29.1)	0 (0)	
5 doses	1 (2)	1 (2.1)	0 (0)	
External beam radiotherapy (No)	0 (0)	0 (0)	0 (0)	**—**
Recurrence (Yes)	11 (9.6)	11 (11.8)	0 (0)	0.47
Survival (Yes)	94 (100) a	91 (100)	3 (100)	**—**

Values expressed as frequency (%) except where stated otherwise. *M* ± *SD* mean ± standard deviation; *PTC* papillary thyroid cancer; *FTC* follicular thyroid cancer; RAI radioactive iodine ablation therapy.

a Computed after excluding 20 patients with missing information; **—** not applicable.

### 3.3 DTC among filipinos by sex

We compared the characteristics of DTC among Filipinos by sex. No significant sex differences were observed across the clinical, ultrasonographic, FNA, recurrence and survival features, as well as across most laboratory variables (data not presented). Most histologic variables did not show sex differences. However, more males displayed positive tumor lymphovascular invasion (52.6%_♂_ vs 18.9%_♀_, *P* = 0.002); presented with >1 tumor nodule (63.2%_♂_ vs 40.7%_♀_, *P* = 0.03); had stage 4 disease (10.5%_♂_ vs 0%_♀_ *P* = 0.005); and metastases (11.1%_♂_ vs 1.1%_♀_, *P* = 0.02). These findings suggested that in Qatar, Filipino males encountered a more aggressive disease than females.

### 3.4 DTC among filipinos living outside the Philippines: Qatar vs Canada

Table 7 (Section A) depicts the comparison of our findings with those of Filipinos with DTC living in Canada [7]. DTC behaviour among both patient populations was very similar, with the exception that in Canada, significantly more patients received radiotherapy (EBRT) after the initial surgery compared to Qatar. This suggested that DTC among Filipinos in both countries appeared to be fairly homogenous.

**Table 7:** Filipinos diagnosed with DTC living outside and in the Philippines based on international studies.

Variable	A. Outside Philippines		B. In Philippines			*P*^f^	*P*^g^	*P*^h^	*P*^i^
	Qatar^a^	Canada^b^	Lo et al.^c^	Mendoza et al.^d^	Canete et al.^e^				
	N = 114	N = 36	N = 728	N = 225	N = 420				
Study duration	2015–2021	1984–2003	1990–2014	2007–2011	2008–2011				
Demography									
Sex						0.98	0.47	0.92	0.29
Male	19 (16.7)	6 (16.7)	103 (14.1)	40 (17.8)	89 (21)				
Female	95 (83.3)	30 (83.3)	625 (85.9)	185 (82.2)	331 (79)				
Age (yr, M ± SD)	40.15 ± 9.86		44 ± 13	43.92 ± 12.61	38.68 ± 12.89	**—**	*0.0003*	*0.005*	0.26
Age bracket (yr)						0.24	*0.002*	*0.003*	**—**
< 45	79 (69.3)	21 (58.3)	395 (54.3)	119 (52.9)					
≥ 45	35 (30.7)	15 (41.7)	333 (45.7)	106 (47.1)					
History									
Family history of thyroid cancer/disease	Cancer 5 (4.4)	Cancer 1 (2.8)	Cancer 11 (1.5)		Disease 58 (14)	0.74	0.17	**—**	*0.003*
History: head/neck radiation therapy	0 (0)	0 (0)				**—**	**—**	**—**	**—**
Hyperthyroid symptoms	6 (5.4)				49 (12)	**—**	**—**	**—**	*0.04*
Neck swelling	88 (78.6) j				420 (100)^k^	**—**	**—**	**—**	*<0.0001*
Laboratory (Pre/post op)									
Free T4, pmol/L	13.78 ± 3.06				16.98 ± 6.9	**—**	**—**	**—**	*<0.0001*
TSH, mIU/L	2.38 ± 3.99			2.1 ± 1.47	1.61 ± 0.92	**—**	**—**	*<0.0001*	*0.0003*
Ultrasonography									
Nodule composition						**—**	**—**	**—**	*<0.0001*
Cystic	6 (5.5)				15 (3.5)				
Mixed solid/cystic	23 (20.9)				15 (3.5)				
Solid	81 (73.6)		564 (82.5)		390 (93)		*0.03*		
Micro-calcification (yes)	19 (17.1)		157 (23)		191 (45)	**—**	0.18	**—**	*<0.0001*
Multi-nodularity (yes)	57 (50.9)		542 (79.2)			**—**	*<0.0001*	**—**	**—**
Maximum size (cm)	2.60 ± 1.32		3.3 ± 2.1			**—**	*<0.0001*	**—**	**—**
FNA Bethesda score						**—**	*<0.0001*	**—**	**—**l
I	1 (0.9)		0 (0)						
II	16 (14.7)		325 (46.3)		180 (43)^m^				
III	28 (25.7)		2 (0.3)						
IV	2 (1.8)		26 (3.7)		12 (3)^m^				
V	14 (12.8)		67 (9.6)		156 (37)^m^				
VI	48 (44.1)		282 (40.1)		72 (17)^m^				
Initial thyroid surgery						0.786	**—**	**—**	**—**
Total thyroidectomy	64 (56.1)	22 (61.1)							
Hemi/subtotal thyroidectomy	18 (15.7)	6 (16.7)							
Total plus neck dissection^n^	32 (28.1)	8 (22.2)							
Nodal/neck dissection						**—**			
Near total thyroidectomy						**—**			
Postsurgical hypoparathyroidism	4 (3.5)		51 (7)			**—**	0.153	**—**	**—**
Histopathology									
Tumor pathologic findings						0.54	*0.008*	**—**	**—**
FTC	4 (3.5)	3 (8.3)	79 (10.9)						
PTC	110 (96.5)	30 (83.3)	649 (89.1)						
Tumor size (cm, M±SD)	2.31 ± 1.54		2.96 ± 1.87		4.53 ± 2.5	**—**	*0.01*	**—**	<0.0001
Tumor size (cm)						0.35	**—**	0.74^o^	**—**
< 4	94 (84.7)	28 (77.8)		188 (83.6)					
≥ 4	17 (15.3)	8 (22.2)		37 (16.4)					
Tumor multifocality	51 (45.9)	13 (36.1)	158 (21.7)	64 (28.4)	134 (32)	0.37	*<0.0001*	*0.003*	*0.012*
Low risk PTC variant	103 (93.6)			213 (94.7)		**—**	**—**	0.70	**—**
LV invasion (yes)	27 (24.8)			39 (17.3)		**—**	**—**	0.13	**—**
Staging						**—**	*<0.0001*	**—**	**—**
T									
T1	49 (43.8)		248 (34.1)						
T2	28 (25.0)		304 (41.6)						
T3	35 (31.3)		126 (17.4)						
T4	0 (0)		50 (6.9)						
N						**—**	*<0.0001*	*<0.0001*	**—**
NX	43 (37.7)		0 (0)	0 (0)					
N0	28 (24.6)		528 (72.5)	143 (63.6)					
N1a	20 (17.5)		155 (21.3)	18 (8)					
N1b	21 (18.4)		122 (6.2)	64 (28.4)					
M						**—**	*0.041*	**—**	**—**
M0	114 (100)		694 (95.3)						
M1	0 (0)		34 (4.7)						
Stages (AJCC) p						0.124	*<0.0001*	*<0.0001*	**—**
Stage I	97 (85.1)	28 (77.8)	453 (62.2)	146 (65)					
Stage II	11 (9.6)	2 (5.6)	90 (12.3)	21 (9.3)					
Stage III	4 (3.5)	5 (13.9)	106 (14.6)	12 (5.3)					
Stage IV	2 (1.8)	1 (2.8)	79 (10.9)	46 (20.4)					
Post-operative management									
Initial RAI (yes)	75 (74.3)	31 (86.1)	523 (71.8)			0.23	0.190	**—**	**—**
Initial EBRT (yes)	0 (0)	3 (8.3)	13 (2.1)			*0.047*	0.126	**—**	**—**

Values expressed as frequency (%) except where stated otherwise. *M* ± *SD* mean ± standard deviation.; *FNA* fine needle aspiration cytology; *EBRT* external beam radiation therapy; *LV* lympho vascular; RAI radioactive iodine therapy; *PTC* papillary thyroid carcinoma; *FTC* follicular thyroid cancer; *yr* year; *AJCC* American Joint Committee on Cancer; FNAC Bethesda score (I Nondiagnostic or Unsatisfactory, II Benign, III Atypia or FLUS, IV Follicular Neoplasm or Suspicious for a Follicular Neoplasm, V. Suspicious for Malignancy, VI Malignant); **—** not applicable; Italicized cells indicate statistical significance.

a Current study, Hamad General Hospital, Doha, Qatar, Data in this column represent the whole cohort of DTC patients (PTC + FTC).

b Kus et al. [7], 36 patients diagnosed with thyroid cancer, Mount Sinai Hospital tertiary referral center in Toronto, Ontario, Canada.

c Lo et al. [11], 728 patients diagnosed with DTC at Philippine General Hospital, Manila.

d Mendoza et al. [22], 225 PTC patients at University of Santo Tomas Hospital, Manila, Philippines. Hence, we only used the data of our PTC patients (from Table 1, Table 2) when running our comparisons with this study.

e Canete et al. [21], 420 patients diagnosed with malignant thyroid tumors at Philippine General Hospital, Manila, Philippines.

f Kus et al. [7] vs current study.

g Lo et al. [11] vs current study.

h Mendoza et al. [22] vs current study.

i Canete et al. [21] vs current study.

j Based on patient-reported history.

k Based on clinical examination.

l Comparison not undertaken as score was based on Papanicolaou Society of Cytopathology for FNA procedure and reporting.

m Based on Papanicolaou Society of Cytopathology for FNA procedure and reporting. For descriptive purposes only, Canete et al. [21] reported the categories benign/colloid, follicular neoplasm, suspicious for malignancy, and malignant which we placed against our Bethesda stages II, IV, V, and VI.

n Total plus any type of neck or mediastinal lymph node dissection.

o We recomputed our data using the cutoffs employed by Mendoza et al. [22] (≤4 and > 4 cm) and then ran the comparisons.

p Current study and all other studies used for comparisons employed the AJCC staging, except Kus et al. [7] who used deGroot et al. [42] staging.

### 3.5 DTC among filipinos living outside vs in the Philippines: Qatar vs Philippines

The above picture differed when we compared our findings with three studies of Filipino DTC patients in mainland Philippines [11,21,22]. Table 7 (Section B) suggests that although sex distribution was similar between Qatar vs the Philippine studies [11,21,22]; the epidemiology appeared to be quite dissimilar.

As for age at presentation, Qatar patients were slightly younger than those of two Philippine studies [11,22]. In connection with clinical and laboratory findings, the Philippines exhibited more family history of thyroid disease, and history of thyrotoxicity and neck swelling at presentation, supporting that in the same study [21], mean preop TSH was significantly lower, while mean T4 level was significantly higher than in Qatar (although all levels were within normal range). The Philippines had also higher prevalence FTC [11], solid nodules [11,21], micro-calcifications [21], larger tumors and multi-nodular disease [11]. Histopathology confirmed that in the Philippines, tumors were significantly larger, and displayed lower prevalence of AJCC stage I, as well as higher prevalence of stage IV disease [11,22]. Similarly, TNM classification showed significant differences in the N categories [11,22], and significantly more M1 patients in the Philippines vs Qatar (4.7% vs 0%) [11]. Tumor multifocality was significantly more in Qatar [11,21,22], and there were significant differences in FNA findings between Qatar and Philippines [11].

## 4 Discussion

DTC is the most common endocrine malignancy [26]. To the best of our knowledge, the current study is the first to appraise DTC among Filipinos living in the MENA region and the first to undertake in-depth comparisons of such findings to Filipino patients diagnosed with DTC living in and outside the Philippines. The main findings were that the behaviour and epidemiology of DTC among Filipinos living Qatar vs Canada appears to be homogenous, with considerable similarities and overlap. Conversely, DTC among Filipinos living Qatar vs the Philippines appears to be heterogenous: Qatar had younger patients, less thyrotoxicity, neck swellings, solid nodules, micro-calcifications, multi-nodularity; smaller tumors; more stage I and less stage IV disease; and no distant metastasis (*P* 0.01 to < 0.0001). The mean follow up of our patients was 2.61 ± 1.82 years, with the maximum follow up being 6.84 years.

During the study period, DTC incidence was ≈43.84 cases/100,000 Filipinos living in Qatar. Thyroid cancer incidence among Filipino patients aged ≥20 years in the USA was 19.57/100,000 Filipinos [27]. Our observed higher rate might be due to that: thyroid cancer affects young/middle-aged persons, and Filipino expats in Qatar are middle aged [28]; we examined Qatar during 2015–2020 while the US study [27] reported on 2004–2014, and the frequency of DTC has risen in the past decades [2]; and, Qatar offers free healthcare coverage (higher likelihood that all cases would be identified early). Globally, TC incidence based on population-based cancer registries (26 countries) was 47 per 100,000 women and 14 per 100,000 men [4].

Across our Qatar sample, relatively more females had DTC, supporting others [29]. However, DTC among our males exhibited relatively more aggressive features (lymphovascular invasion, number of tumors, stage 4 disease, metastases) than among females. Such higher aggressiveness among males is congruent with other studies [30] and propositions of sex difference in TC pathogenesis [31].

We then compared the DTC characteristics amongst Filipinos living in Qatar and elsewhere. Table 7 (Section A) compares Qatar vs Canada and suggests that DTC epidemiology among Filipinos in Qatar appeared to be fairly homogenous to that in Canada. While it is difficult to speculate the reasons for the observed similarities, a common feature between the Filipino patients with DTC in Qatar and Canada is their immigrant status. Generally, most persons who leave their native country to immigrate or reside in another country for economic betterment are usually healthy individuals (healthy migrant effect). For instance, immigrants were relatively healthier on arrival in Canada compared to native-born Canadians, and immigrant health converged with years in Canada to native-born levels [32]. Commonly, at immigration, migrants have better health status than the remaining population in their native country of birth, and to some extent, better health than the population in the host country [33].

Table 7 (Section B) compares Qatar with three studies of Filipino DTC patients living in mainland Philippines [11,21,22]. Contrary to the similarities we noticed above in DTC behaviour among Filipinos in Qatar and in Canada, the emerging picture was different. With the exception of the sex distribution, DTC epidemiology among Filipinos in Qatar vs the Philippines appeared to be quite heterogenous as discussed below.

Females were more represented in Qatar and in the three Philippine studies, supporting a ratio of 4.5:1 reported by other 10.13039/100006922thryoid cancer research in the Philippines [29,34]. Over-representation of females is well illustrated in our sample, despite that Qatar's demography has high male proportion [17]. As for age at presentation, mean age of the Qatar sample was slightly younger than in the Philippine studies [11,22], probably due to the young/middle-aged demography of expats in Qatar [17].

Clinically, the Philippines had more family history of thyroid disease, hyperthyroid symptoms and neck swelling at presentation than Qatar [21]. Whilst it is difficult to speculate why, the prevalence of iodine deficiency might offer clues. Iodine deficiency is not a health concern in Qatar [35]; while many regions in the Philippines remain iodine deficient [36], although some reports maintain that the Philippines had overcome its iodine deficiency [22]. Lack of dietary iodine is a cause of thyroid disorders, but excess iodine, genetics, geographical and dietary factors also trigger thyroid disorders [37]. Hence, others proposed environmental effects due to exposure to a carcinogenic agent in volcanic lava [38]. If this was the case, Filipinos living outside the Philippines (e.g., Qatar) might be less exposed. Such lack of exposure to environmental effect leading to a decreased risk of thryoid cancer is recognized [39]. Still others suggested that genetic profiles might be the cause [40].

In addition, some of our findings suggested that DTC in the Philippines is more advanced at presentation: ultrasound showed more solid nodules, micro-calcifications, larger tumors, and multi-nodular disease than observed in Qatar, features that suggest thryoid cancer risk or development of multiple recurrences [8,41]; patients presented with higher tumor burden (less stage I, more stage IV disease) [11,22]; and TNM classification showed that 4.7% of patients in the Philippines had distant metastasis (M1) [11] vs 0% in Qatar. Collectively, the above clinical, ultrasound and histopathology findings suggested that DTC among Filipinos in Qatar appears to behave in a quite dissimilar manner compared to that in the Philippines.

### 4.1 Clinical implications

The less suspicious disease presentation and initial ultrasonographic findings in Qatar compared to the Philippines might sometimes lead the unsuspecting health worker to erroneously misinterpret it as non-malignant disease. DTC in Qatar displayed significantly higher proportions of multifocal tumors not readily detected by initial US. Hence, a high index of suspicion is required among Filipinos living in Qatar with thyroid nodules, even with small nodules. This warrants the implementation of additional multi-fold opportunistic strategies for earlier detection among this population group: education of Filipinos on thyroid self-examination; raising awareness/training of primary/health care professionals to undertake thorough thyroid examination during routine checkups of Filipinos; and to assess any suspicion of thyroid nodules or malignancy when Filipinos have routine imaging scans for other health problems.

### 4.2 Limitations and strengths of study

This study has limitations. A larger sample size would have beneficial to test differences between PTC and FTC patients. Retrospective medical record reviews have limitations of data quality. For about 17.5% of the sample, we had no information on survival, probably because they left Qatar. Comparisons based on specific genetic and molecular markers would have been beneficial. The comparisons of our sample with three Philippine studies [11,21,22] or the study in Canada [7] were limited by the different reporting of the disease characteristics across the published papers; consensus and standardization of the reporting of thyroid cancer would benefit similar comparisons across different countries. Unpacking the reasons behind the differences between Filipino patients with DTC living in Philippines vs outside the Philippines is not a straightforward one. Future research could benefit from exploring such limitations.

Nevertheless, the study has many strengths. To our knowledge, it is the first to appraise DTC among Filipinos living in the MENA region, employing many variables. In addition, it is representative of the Filipino population living in Qatar as our institution receives all cases across the country. We undertook the rare detailed comparison of Filipino patients with DTC in Qatar and the Philippines, employing three suitable and different studies undertaken in the Philippines involving data stretching over more than two decades (24 years, 1990–2014), and including a large number of (1373) of DTC patients seen at 3 different main tertiary care hospitals at various locations in Manila. We are not aware of any previous study that undertook such analysis.

## 5 Conclusion

The epidemiology of DTC among Filipinos living Qatar vs Canada appears to be homogenous, with considerable similarities and overlap. Conversely, DTC among Filipinos living Qatar vs the Philippines appears to be heterogenous. It appears that DTC among Filipinos living in Qatar has presentations that could erroneously be misinterpreted as non-malignant by the unsuspecting health worker, and seemingly lower tumor load compared to those living in the Philippines. Hence, thyroid lesions among Filipinos in Qatar should be taken very seriously. These characteristics warrant the implementation of additional opportunistic practices for earlier detection among this population group. Data on DTC among Filipinos living in the MENA region are scarce; surgeons and endocrinologists should be encouraged to publish such data.

## Funding

This research did not receive any specific grant from funding agencies in the public, commercial, or not-for-profit sectors.

## Competing interest

The authors of this manuscript have no conflicts of interest to declare. All co-authors have seen and agree with the contents of the manuscript and there is no financial interest to report. We certify that the submission is original work and is not under review for other publication.

## Provenance and peer review

Not commissioned, externally peer-reviewed.

## CRediT authorship contribution statement

**Mohamed Said Ghali:** Conceptualization, Methodology, Investigation, Data curation, Writing – original draft, Writing – review & editing. **Walid El Ansari:** Conceptualization, Methodology, Investigation, Data curation, Writing – original draft, Writing – review & editing. **Abdelrahman Abdelaal:** Conceptualization, Methodology, Writing – review & editing. **Mohamed S. Al Hassan:** Conceptualization, Methodology, Writing – review & editing.

## References

[1] Dong W., Horiuchi K., Tokumitsu H. (2019). Time-varying pattern of mortality and recurrence from papillary thyroid cancer: lessons from a long-term follow-up. Thyroid.

[2] Lo T.E., Canto A.U., Maningat P.D. (2015). Risk factors for recurrence in Filipinos with well-differentiated thyroid cancer. Endocrinol Metab (Seoul).

[3] Parameswaran R., Shulin H.J., Min E.N., Tan W.B., Yuan N.K. (2017). Patterns of metastasis in follicular thyroid carcinoma and the difference between early and delayed presentation. Ann. R. Coll. Surg. Engl..

[4] Li M., Dal Maso L., Vaccarella S. (2020). Global trends in thyroid cancer incidence and the impact of overdiagnosis. Lancet Diabetes Endocrinol..

[5] Lundgren C.I., Hall P., Dickman P.W., Zedenius J. (2007). Influence of surgical and postoperative treatment on survival in differentiated thyroid cancer. Br. J. Surg..

[6] Clark J.R., Eski S.J., Freeman J.L. (2006). Risk of malignancy in Filipinos with thyroid nodules-a matched pair analysis. Head Neck.

[7] Kus L.H., Shah M., Eski S., Walfish P.G., Freeman J.L. (2010). Thyroid cancer outcomes in Filipino patients. Arch. Otolaryngol. Head Neck Surg..

[8] Palme C.E., Waseem Z., Raza S.N., Eski S., Walfish P., Freeman J.L. (2004). Management and outcome of recurrent well-differentiated thyroid carcinoma. Arch. Otolaryngol. Head Neck Surg..

[9] Haugen B.R., Alexander E.K., Bible K.C. (2015). American thyroid association management guidelines for adult patients with thyroid nodules and differentiated thyroid cancer: the American thyroid association guidelines task force on thyroid nodules and differentiated thyroid cancer. Thyroid.

[10] Termorshuizen F., van der Ven E., Tarricone I. (2020). The incidence of psychotic disorders among migrants and minority ethnic groups in Europe: findings from the multinational EU-GEI study [published online ahead of print, 2020 Sep 22]. Psychol. Med..

[11] Lo T.E., Uy A.T., Maningat P.D. (2016). Well-differentiated thyroid cancer: the philippine general hospital experience. Endocrinol Metab (Seoul).

[12] Jauculan M.C., Buenaluz-Sedurante M., Jimeno C.A. (2016). Risk factors associated with disease recurrence among patients with low-risk papillary thyroid cancer treated at the university of the Philippines-philippine general hospital. Endocrinol Metab (Seoul).

[13] Nguyen M.T., Hu J., Hastings K.G. (2017). Thyroid cancer mortality is higher in Filipinos in the United States: an analysis using national mortality records from 2003 through 2012. Cancer.

[14] Megwalu U.C., Ma Y., Osazuwa-Peters N., Orloff L.A. (2021). Clinical presentation and survival outcomes of well-differentiated thyroid cancer in Filipinos. Cancer Med..

[15] Rodriguez R. (2011). Philippine migrant workers' transnationalism in the Middle East. Int. Labor Work. Class Hist..

[16] Deng Y., Li H., Wang M. (2020). Global burden of thyroid cancer from 1990 to 2017. JAMA Netw. Open.

[17] World population review. https://worldpopulationreview.com/countries/qatar-population.

[18] Tuttle R.M., Haugen B., Perrier N.D. (2017). Updated American Joint committee on cancer/tumor-node-metastasis staging system for differentiated and anaplastic thyroid cancer (eighth edition): what changed and why?. Thyroid.

[19] Lamartina L., Grani G., Arvat E. (2018). 8th edition of the AJCC/TNM staging system of thyroid cancer: what to expect (ITCO#2). Endocr. Relat. Cancer.

[20] Momesso D.P., Tuttle R.M. (2014). Update on differentiated thyroid cancer staging. Endocrinol Metab. Clin. N. Am..

[21] Canete E.J., Sison-Pena C.M., Jimeno C.A. (2014). Clinicopathological, biochemical, and sonographic features of thyroid nodule predictive of malignancy among adult Filipino patients in a tertiary hospital in the Philippines. Endocrinol Metab (Seoul).

[22] Mendoza E.S., Lopez A.A., Valdez V.A. (2016). Predictors of incomplete response to therapy among Filipino patients with papillary thyroid cancer in a tertiary hospital. J. Endocrinol. Invest..

[23] Mathew G., Agha R. (2021). For the STROCSS Group, STROCSS 2021: strengthening the Reporting of cohort, cross-sectional and case-control studies in Surgery. Int. J. Surg..

[24] Researchregistry8011. https://www.researchregistry.com/browse-the-registry#home/registrationdetails/62aae06be8a6c2002198b32f/.

[25] OpenEpi collection of epidemiologic calculators. https://www.openepi.com/Menu/OE_Menu.htm.

[26] Alvarado R., Sywak M.S., Delbridge L., Sidhu S.B. (2009). Central lymph node dissection as a secondary procedure for papillary thyroid cancer: is there added morbidity?. Surgery.

[27] Megwalu U.C., Osazuwa-Peters N., Moon P., Palaniappan L.P. (2021). Thyroid cancer incidence trends among Filipinos in the United States. Laryngoscope.

[28] Alrouh H., Ismail A., Cheema S. (2013). Demographic and health indicators in Gulf Cooperation Council nations with an emphasis on Qatar. J Local Glob Heal Perspect.

[29] Santiago A.G., Isidro M.J., Parra J. (2021). Predictors of response to therapy among post thyroidectomy adult Filipino patients with papillary thyroid carcinoma based on the 2015 American thyroid association guidelines. J ASEAN Fed Endocr Soc.

[30] Shobab L., Burman K.D., Wartofsky L. (2022). Sex differences in differentiated thyroid cancer. Thyroid.

[31] Costa C., Souteiro P., Paredes S. (2022). Male gender as a poor prognostic factor in Medullary Thyroid Carcinoma: behaviour or biological difference?. Minerva Endocrinol..

[32] McDonald J.T., Kennedy S. (2004). Insights into the 'healthy immigrant effect': health status and health service use of immigrants to Canada. Soc. Sci. Med..

[33] Helgesson M., Johansson B., Nordquist T. (2019). Healthy migrant effect in the Swedish context: a register-based, longitudinal cohort study. BMJ Open.

[34] Ong-Ramos C.C., Sawadjaan L., Villa Michael L. (2014). Endocrine malignancies: a five-year retrospective analysis in a tertiary hospital. Philippine J. Intern. Med..

[35] Supreme Council of Health Qatar free of iodine deficiency disorders. https://healthcare.qa/pages/the-sch-qatar-free-of-iodine-deficiency-disorders-idd.

[36] Carlos-Raboca J., Jimeno C.A., Kho S.A. (2014). The philippine thyroid diseases study (PhilTiDeS 1): prevalence of thyroid disorders among adults in the Philippines. J ASEAN Fed Endocr Soc.

[37] Knudsen N., Laurberg P., Perrild H., Bülow I., Ovesen L., Jørgensen T. (2002). Risk factors for goiter and thyroid nodules. Thyroid.

[38] Duntas L.H., Doumas C. (2009). The 'rings of fire' and thyroid cancer. Hormones (Basel).

[39] Rossing M.A., Schwartz S.M., Weiss N.S. (1995). Thyroid cancer incidence in Asian migrants to the United States and their descendants. Cancer Causes Control.

[40] Espiritu G.A.M., Malana J.T., Dumasis A.J.G.V., Ang D.C. (2019). High preponderance of braf V600E mutation in papillary thyroid carcinoma among Filipinos: a clinicopathologic study. J Glob Oncol.

[41] Håskjold O.I., Foshaug H.S., Iversen T.B., Kjøren H.C., Brun V.H. (2021). Prediction of thyroid nodule histopathology by expert ultrasound evaluation. Endocr Connect.

[42] DeGroot L.J., Kaplan E.L., McCormick M., Straus F.H. (1990). Natural history, treatment, and course of papillary thyroid carcinoma. J. Clin. Endocrinol. Metab..

